# Enforcing GLUT3 expression in CD8^+^ T cells improves fitness and tumor control by promoting glucose uptake and energy storage

**DOI:** 10.3389/fimmu.2022.976628

**Published:** 2022-09-20

**Authors:** Elisabetta Cribioli, Greta Maria Paola Giordano Attianese, Pierpaolo Ginefra, Amandine Signorino-Gelo, Romain Vuillefroy de Silly, Nicola Vannini, Christoph Hess, Melita Irving, George Coukos

**Affiliations:** ^1^ Ludwig Institute for Cancer Research, University of Lausanne, Lausanne, Switzerland; ^2^ Department of Oncology, University of Lausanne and Lausanne University Hospital Centre Hospitalier Universitaire Vaudois (CHUV), Lausanne, Switzerland; ^3^ Department of Biomedicine, Immunobiology, University of Basel and University Hospital of Basel, Basel, Switzerland; ^4^ Department of Medicine, Cambridge Institute of Therapeutic Immunology & Infectious Disease (CITIID), Jeffrey Cheah Biomedical Centre, University of Cambridge, Cambridge, United Kingdom

**Keywords:** T cell engineering, metabolism, glucose, immunotherapy, tumor, adoptive cell therapy

## Abstract

Despite the tremendous success of adoptive T-cell therapies (ACT) in fighting certain hematologic malignancies, not all patients respond, a proportion experience relapse, and effective ACT of most solid tumors remains elusive. In order to improve responses to ACT suppressive barriers in the solid tumor microenvironment (TME) including insufficient nutrient availability must be overcome. Here we explored how enforced expression of the high-affinity glucose transporter GLUT3 impacted tumor-directed T cells. Overexpression of GLUT3 in primary murine CD8^+^ T cells enhanced glucose uptake and increased glycogen and fatty acid storage, and was associated with increased mitochondrial fitness, reduced ROS levels, higher abundance of the anti-apoptotic protein Mcl-1, and better resistance to stress. Importantly, GLUT3-OT1 T cells conferred superior control of B16-OVA melanoma tumors and, in this same model, significantly improved survival. Moreover, a proportion of treated mice were cured and protected from re-challenge, indicative of long-term T cell persistence and memory formation. Enforcing expression of GLUT3 is thus a promising strategy to improve metabolic fitness and sustaining CD8^+^ T cell effector function in the context of ACT.

## Introduction

Cancer-specific T cells are key to the immune control of tumors. Circulating naïve cells are typically primed in the tumor draining lymph nodes by professional antigen presenting cells (APCs), from where they migrate into the tumor to exert their effector functions (1). During this process, T cells shift from a metabolically quiescent state reliant upon mitochondrial oxidative phosphorylation (OXPHOS) and fatty acid oxidation (FAO) (2), to an activated effector state heavily dependent upon aerobic glycolysis (3–7). This metabolic switch is critical as it enables T cells to generate biosynthetic precursors needed to support effector functions (4) and clonal expansion (8). In this process of effector maturation T cells also rapidly upregulate the expression of nutrient transporters including those facilitating import of glucose (8–10). Memory T cells, which emerge upon antigen clearance, are again quiescent and have a metabolic phenotype similar to naïve cells (11).

In the TME, T cells compete for nutrients with tumor cells (12, 13) which can hinder the efficacy of immunotherapy (14, 15). A variety of approaches have been tested to improve metabolic fitness of T cells for ACT, including the optimization of culture conditions and various gene-modifications (5). Ideally, culture conditions allow sufficient expansion of the T cell product for ACT while minimizing differentiation [i.e., promoting stem-cell like (T_SMC_) and central memory (T_CM_) T cell phenotypes] to allow optimal engraftment/persistence which is critical for tumor control in patients (16).

GLUT1 is the most highly studied glucose transporter in immune cells, however, particularly in CD8^+^ T cells, a prominent role for high affinity glucose transporter, GLUT3, has recently been suggested (9, 10). GLUT3 has both a higher affinity for glucose and increased transport capacity than GLUT1, which may explain its predominant expression within the central nervous system (17) and other highly glucose-dependent tissues (18). Based on its functional characteristics we set out to explore GLUT3 as a candidate for metabolic bioengineering in the context of solid tumor-targeting ACT.

## Results

### Enforcing expression of GLUT3 drives glucose uptake by T_EM_ cells

**Figure 1:**
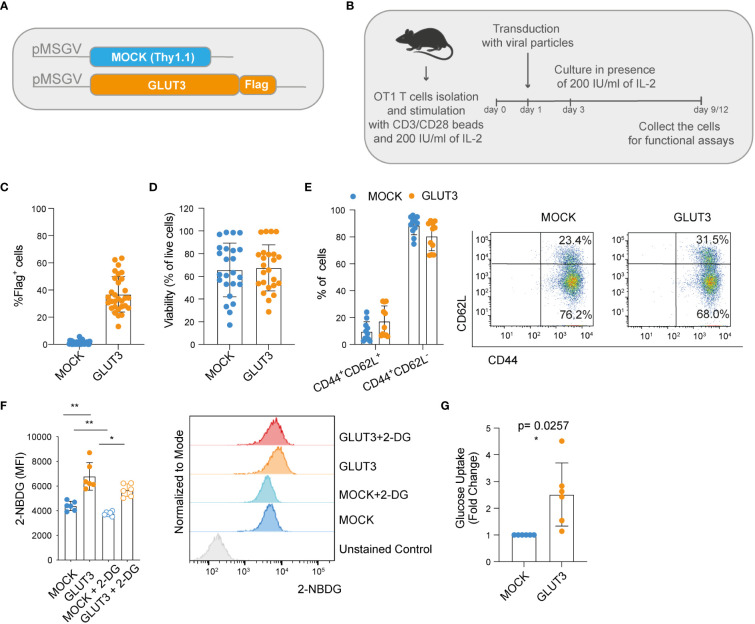
Enforcing expression of GLUT3 enhances glucose uptake by T_EM_ cells. **(A)** Schematic of pMSGV retroviral vectors encoding Thy1.1 (T cell engineering control termed MOCK), and GLUT3 comprising a Flag tag. **(B)** Schematic of OT1 T cell isolation, activation and expansion in high-dose IL-2. **(C)** Evaluation of transduction efficiency for GLUT3 expression by anti-Flag antibody staining and flow cytometric analysis. **(D)** Viability of transduced T cells as measured by staining with a viability dye and flow cytometric analysis (days 9-12). **(E)** Percentage of CD44^+^ CD62L^+^ and CD44^+^ CD62L^-^ T cells in culture. Left: summary of all donors tested. Right: representative flow cytometry dot plot. **(F)** Left: glucose uptake by T cells evaluated with the fluorescent glucose analog 2-NBDG with or without the addition of the glucose competitor 2-DG. Right: representative histograms showing 2-NBDG uptake. **(G)** Glucose uptake by T cells measured in a luminescence-based assay. Shown is average ± standard deviation (SD) of different cultures. For data in which fold-change is shown, values of GLUT3 expressing T cells of each culture are normalized with the values of the corresponding MOCK-T cells. Shown is average ± standard deviation (SD) of different cultures. Statistical analysis by paired, two-tailed t test (**F**: MOCK vs GLUT3 p=0.001, MOCK vs MOCK+2DG p= 0.0029, GLUT3 vs a GLUT3 +2DG p=0.0138). **p< 0.01; *p < 0.05.

We began by building retroviral vectors encoding the marker Thy1.1 (control MOCK-T cells), as well as codon-optimized GLUT3 comprising a Flag tag for detection purposes (**Figure 1A**). Splenic derived OT1 CD8^+^ T cells were activated with anti-CD3/CD28 beads, transduced the following day, and then expanded in the presence of 200 IU/ml of IL-2 for 9-12 days to generate predominantly effector memory T cells (GLUT3-T_EM_ cells; CD44^+^CD62L^–^) (**Figure 1B**). Transduction efficiencies for GLUT3 were variable amongst donors, ranging from 10% to over 60% (**Figure 1C**). In comparison, Thy1.1 varied between ~40-60% transduction efficiency (MOCK-T_EM_ cells, **Figure S1A**). To account for this variability, when appropriate, comparisons between GLUT3-T cells and MOCK-T cells are shown normalized to values obtained in the mock-transfected population from the same donor.

We observed no differences in viability between the MOCK- and GLUT3-T cells (**Figure 1D**), nor in phenotype following expansion (**Figure 1E**). To estimate glucose uptake, 2-(N-(7-Nitrobenz-2-oxa-1,3-diazol-4-yl) Amino)-2-Deoxyglucose (2-NBDG) uptake experiments were performed. 2-NBDG uptake was significantly higher for GLUT3- over MOCK-T cells and attenuated in the presence of 2-deoxy D-glucose (2-DG) in both subsets (**Figure 1F**). Higher glucose uptake by GLUT3-T cells, in comparison to MOCK transduced cells, was further validated in a luminescence-based assay (**Figure 1G**).

Together these data demonstrated that CD8^+^ T cells could be retrovirally engineered to overexpress GLUT3, enabling increased glucose uptake in the absence of alterations to phenotype or *in vitro* cellular expansion.

### Enforcing expression of GLUT3 promotes energy storage by T_EM_ cells

**Figure 2:**
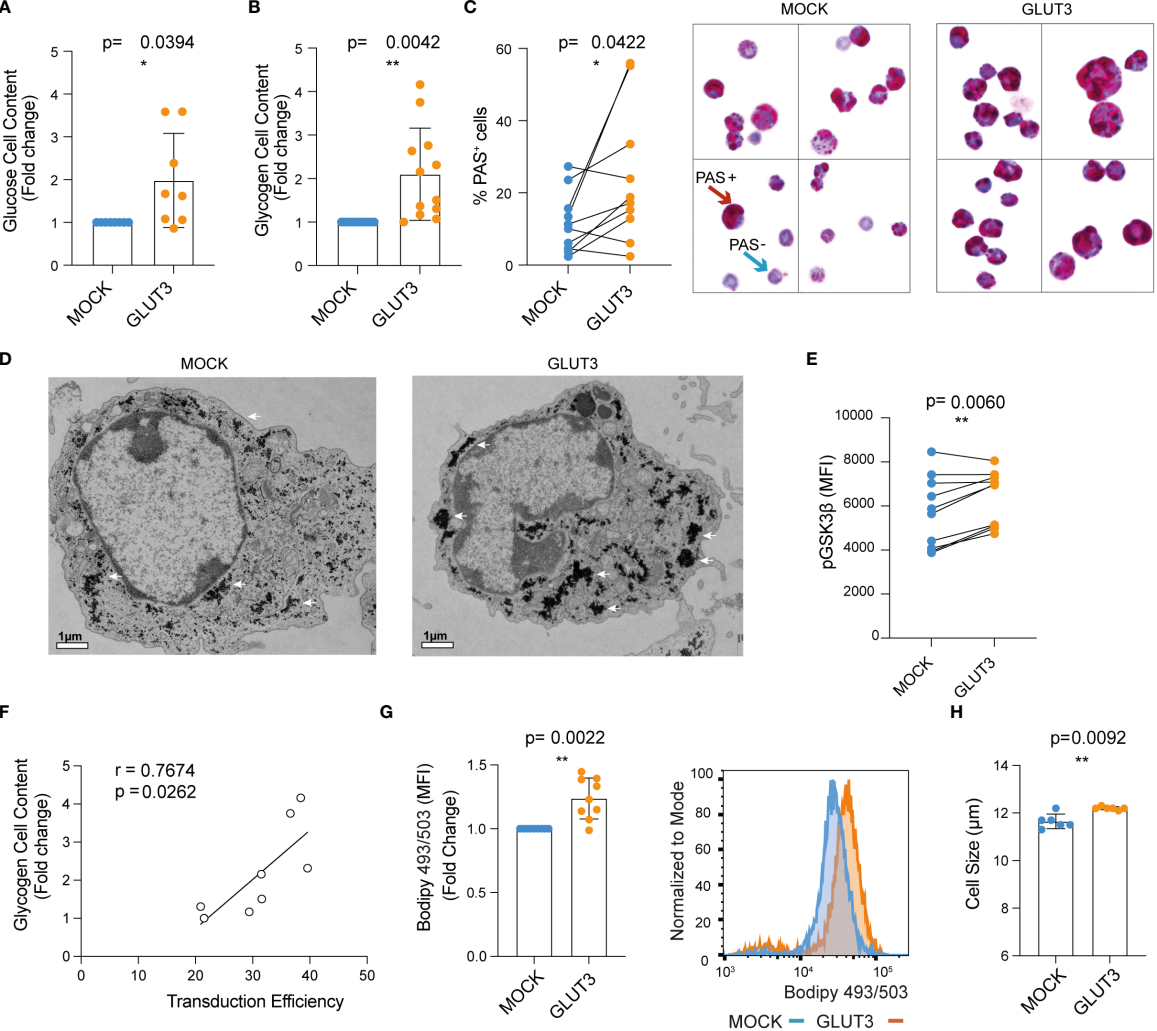
Enforcing expression of GLUT3 is associated with elevated energy storage by T_EM_ cells. **(A)** Glucose content in T cell lysate measured with a colorimetric assay. **(B)** Glycogen content in T cells as evaluated with a colorimetric assay. **(C)** Left: summary of periodic acid shiff (PAS)-based evaluation of glycogen in MOCK- versus GLUT3-T cells. Right: representative 100X images of PAS staining of MOCK- versus GLUT3-T cells. Red arrow indicates an example of a PAS positive cell (magenta), blue arrow indicates example of a negative cell (purple, hematoxylin positive). **(D)** Representative transmission electron microscopy pictures (magnification 3800X) of MOCK- versus GLUT3-T cells. White arrows point to glycogen deposits. **(E)** Intracellular staining for pGSK3β (at Ser9). **(F)** Correlation between glycogen content and percentage of transduction. **(G)** Left : mean fluorescence intensity (MFI) for T cells stained with Bodipy 493/503 dye. Right: representative histogram of Bodipy 493/503 dye staining. **(H)** Size of MOCK- versus GLUT3-T cells (μm). For data in which fold-change is shown, values of GLUT3 expressing T cells of each culture are normalized with the values of the corresponding MOCK-T cells. Shown is average ± SD of different cultures. Statistical analysis by paired, two-tailed t test **(A–C, E, G, H)**, Pearson correlation (r=correlation coefficient) **(F)**. **p< 0.01; *p < 0.05.

Given the increased uptake of glucose in GLUT3 overexpressing CD8^+^ T cells, we next evaluated how this surplus in energy was stored. On one hand, a significant increase in cellular glucose content for GLUT3- versus MOCK-T cells was observed (**Figure 2A**). In addition, glycogen was more abundant in GLUT3-T cells (**Figures 2B, C**, and **Figures S1B, C**) (19). Increased glycogen content in GLUT3-T cells was further validated by transmission electron microscopy (**Figure 2D**).

Glycogen synthase kinase-3 β (GSK-3β), a master serine-threonine kinase, is involved in the synthesis and storage of glycogen in several cell types (20). Phosphorylation at Ser9 inactivates GSK-3β (21) so that it is no longer able to phosphorylate and inactivate glycogen synthase. Notably, it has been shown that GSK-3β inhibition causes nuclear factor of activated T cells 2 (NFAT2) to accumulate in the nucleus of T cells and increases the proliferation of T cells and supports IL-2 production (22). Moreover, GSK-3β inhibition promotes the formation of CD8^+^ memory stem cells (23). Indeed, in our study higher levels of pGSK-3β (at Ser9) in GLUT3- versus MOCK-T cells were observed, further indicative of increased production of glycogen in T cells gene-modified to over-express GLUT3 (**Figure 2E**). Notably, glycogen content of the GLUT3-T cells was directly proportional to transduction efficiency (**Figure 2F**).

Since glucose can also be converted to fatty acids, we further performed Bodipy 493/503 staining and observed significantly higher lipid abundance in GLUT3- over MOCK T cells (**Figure 2G**). Interestingly, GLUT3 overexpression was further associated with an increase in the diameter of cells (**Figure 2H**).

In all, these data established that GLUT3 overexpressing OT1 CD8^+^ T cells were characterized by increased cellular glucose content as well as accumulation of glycogen and lipids. Aligning with these findings, enforced expression of GLUT3 was further associated with inactivation of GSK-3β.

### Enforcing expression of GLUT3 in T_EM_ cells is associated with superior metabolic fitness and effector function

**Figure 3:**
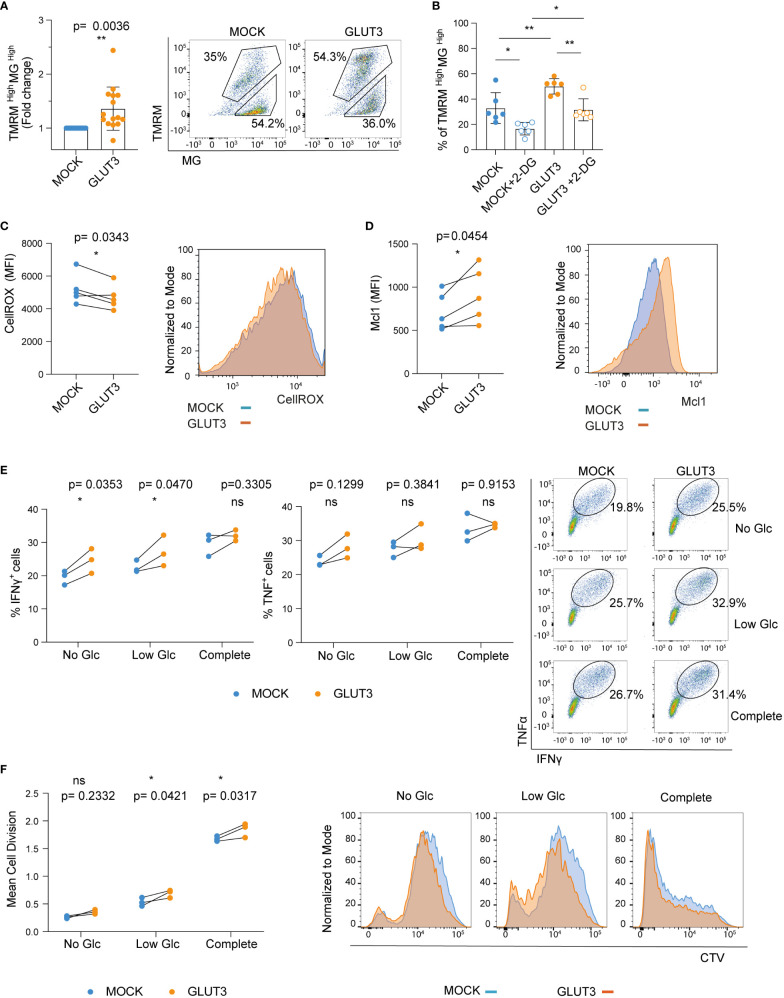
Enforcing expression of GLUT3 is associated with higher mitochondrial polarization and improved *in vitro* function of T_EM_ cells. **(A)** Staining of T cells with TMRM and Mitotracker green (MG). Left: TMRM^high^MG^high^ values for GLUT3-T cells are normalized with the values for MOCK-T cells from the same donor. Right: representative dot plot of TMRM and MG stained T cells. Upper gating represents the TMRM^high^MG^high^ population. **(B)** Percentage of TMRM^high^MG^high^ T cells following overnight incubation with 2-DG **(C)** Left: quantification of intracellular reactive oxygen species (ROS) with CellROX Green reagent. Right: representative plot of CellROX staining. **(D)** Left: evaluation of Mcl-1 levels by intracellular staining. Right: representative plot of anti-Mcl-1 antibody-stained cells. **(E)** Left and middle: Analysis of IFNγ and TNF production by T cells upon overnight stimulation with SIINFEKL peptide in media deprived of glucose (Glc), media with low Glc (Glc= 0.444 mM), or complete media (Glc= 11.1 mM). Right: representative dot plot of anti-IFNγ and anti-TNF antibody staining upon stimulation with SIINFEKL peptide in different media. Shown is one representative experiment out of three. **(F)** Left: mean cell division of T cells stimulated for 5 days with irradiated B16-OVA tumor cells in media deprived of Glc, media with low Glc concentration, or complete media. Right: representative histograms of cell trace violet (CTV) dilution proliferation experiment. Shown is one representative experiment out of three. Shown is average ± SD of different cultures. Statistical analysis by paired, two-tailed t test **(A, C, D)**, two-way analysis of variance (ANOVA) with correction for multiple comparisons by *post hoc* Tukey’s test (**B**: MOCK vs MOCK+2-DG p=0.0131; MOCK vs GLUT3 p=0.0089; GLUT3 vs GLUT3+2-DG p= 0.0049; MOCK+2-DG vs GLUT3+2-DG p=0.0235), two-way ANOVA with correction for multiple comparisons by *post hoc* Sidak’s test **(E, F)**. **p< 0.01; *p < 0.05; ns, non -significant.

We next sought to evaluate the metabolic fitness and *in vitro* effector function of GLUT3-T_EM_ cells. First, we examined mitochondrial membrane potential as well as mass using Tetramethylrhodamine-methyl ester (TMRM) and Mitrotracker Green (MG) staining, respectively. Enforced GLUT3 expression was associated with an increase in the proportion of TMRM^high^MG^high^ T cells (**Figure 3A**). This increase in the proportion of TMRM^high^MG^high^ T cells upon enforced expression of GLUT3 was not observed in the presence of the glucose competitor 2-DG (**Figures 3B**, **S1D**).

It has recently been demonstrated that glycogen metabolism (both its accumulation and degradation) is a critical process for T cell memory formation. Moreover, improved clearance of reactive oxygen species (ROS) is associated with enhanced T cell survival (24). In line with these findings, ROS levels were lower in GLUT3-T cells (**Figure 3C**). In addition, the antiapoptotic protein Mcl-1, which is stabilized in cells with increased glucose metabolism (25), was more abundant in GLUT3-T cells compared to MOCK T cells counterparts (**Figure 3D**).

To evaluate how GLUT3 overexpression related to T cell effector function we stimulated engineered T cells overnight with the ovalbumin SIINFEKL peptide and measured IFNγ and TNF production in the presence of increasing concentrations of glucose. Production of both IFNγ and TNF by GLUT3-T cells tended to be higher than in MOCK T cells (**Figure 3E**). In addition, GLUT3-T cells proliferated better in low glucose media when co-cultured with irradiated B16-OVA tumor cells, a trend also observed in absence of glucose and when using complete media (**Figure 3F**).

Enforced expression of GLUT3 was thus associated with increased mitochondrial polarization, lower levels of ROS, and upregulation of the anti-apoptotic protein Mc1-1. Enhanced cytokine production and proliferation were immune correlates of this metabolic phenotype induced in GLUT3 overexpressing T cells.

### Enforcing expression of GLUT3 in T_CM_ cells improves resistance to cell death and exhaustion

**Figure 4:**
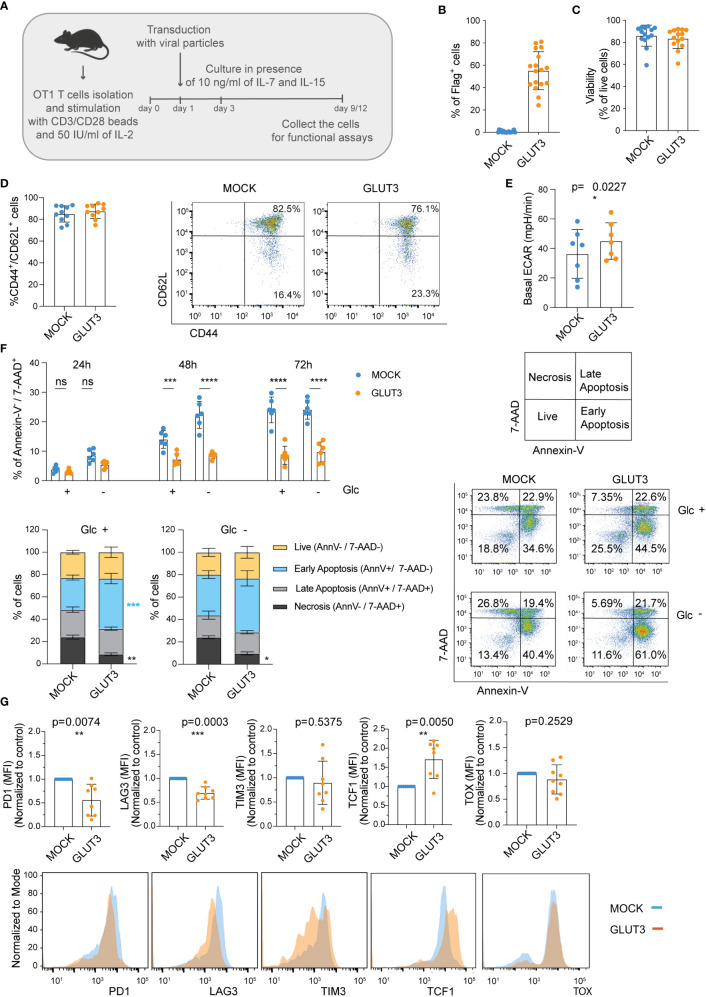
Enforcing expression of GLUT3 enables higher glucose uptake, survival, and resistance to exhaustion of T_CM_ cells. **(A)** Schematic for the generation of GLUT3-T_CM_ cells. **(B)** Evaluation of transduction efficiency for GLUT3 expression by anti-Flag antibody staining and flow cytometric analysis. **(C)** Viability of transduced T cells as measured by staining with a viability dye and flow cytometric analysis (days 9-12). **(D)** Percentage of CD44^+^ CD62L^+^ and CD44^+^ CD62L^-^ T cells in culture. Left: summary of all donors tested. Right: Representative flow cytometry dot plot. **(E)** Seahorse analysis of the basal extracellular acidification rate (ECAR) of MOCK- and GLUT3-T cells with a metabolic perturbation assay. **(F)** Top Left: Percentage necrosis (AnnexinV^-^/7-AAD^+^) of MOCK- versus GLUT3-T cells over time in media with or without glucose (Glc).Bottom Left: evaluation of live (Annexin V^-^/7-AAD^-^), early apoptotic (Annexin V^+^/7-AAD^-^), late apoptotic (Annexin V^+^/7-AAD^+^), and necrotic (Annexin V^-^/7-AAD^+^) MOCK- and GLUT3-T cells after 72h in culture in the presence or absence of Glc. Right: representative dot plot of AnnexinV/7-AAD staining at 72h (AnnV=Annexin V). **(G)** Expression levels of PD1, LAG3, TIM3, TCF-1 and TOX upon repeated stimulation with SIINFEKL peptide for 5 days. Top: mean fluorescence intensity (MFI) levels are shown. MFI levels of GLUT3-T cells from each culture are normalized to corresponding MOCK-T cells. Bottom: representative histograms of PD1, LAG3, TIM3 TCF1 and TOX expression. Shown is average ± SD of different cultures. Statistical analysis by paired, two-tailed *t* test **(E, G)**, two-way analysis of variance (ANOVA) with correction for multiple comparisons by *post hoc* Tukey’s test (**F** Top Left: Glc+ 48h p=0.0005, Glc- 48h p<0.0001, Glc+ 72h p<0.0001, Glc- 72h p<0.0001), two-way ANOVA with correction for multiple comparisons by *post hoc* Sidak’s test (**F** Bottom Left: Glc +, Necrosis MOCK vs GLUT3 p=0.0016; Glc +, Early Apoptosis MOCK vs GLUT3 p=0.0009; Glc -, Necrosis MOCK vs GLUT3 p=0.0456). ****p < 0.0001; ***p < 0.001; **p < 0.01; *p < 0.05; ns, non-significant.

Expansion of murine CD8^+^ T cells in the presence of the common gamma chain cytokines IL-7 and IL-15, as compared to IL-2 only, generates a greater proportion of central memory T cells (T_CM_; CD44^+^CD62L^+^) (schematic in **Figure 4A**). T_CM_ cells are characterized by superior expansion and tumor control *in vivo* (26). Given the higher intake of glucose by GLUT3-T cells, we questioned whether this would limit the generation of T_CM_ cells (27) (i.e., drive effector differentiation) when using a IL-7/IL-15 based expansion protocol. To evaluate this, OT1 T cells were transduced to express GLUT3 (**Figure 4B**) or Thy1.1 (**Figure S2A**) and expanded in the presence of IL-7/IL-15. No differences were noted in viability (**Figure 4C**), cell size (**Figure S2B**), population doubling times (**Figure S2C**), or proportion of T_CM_ (**Figure 4D**) between GLUT3- and MOCK-T cells. Similar to GLUT3-T_EM_ cells, GLUT3-T_CM_ cells were characterized by increased glucose uptake as compared to the MOCK counterpart (**Figure S2D**), and 2-DG attenuated glucose uptake in both cell populations **(** **Figure S2E**). Indicative of more active aerobic glycolysis, basal extracellular acidification rate (ECAR) was higher in GLUT3- than MOCK-T_CM_ cells (**Figure 4E**).

We next tested the idea that increased energy stores acquired by GLUT3-T cells may support their viability *in vitro*. To test this, cells initially expanded in the presence of IL-7/IL-15 were subjected to cytokine deprivation for 24-72 hours *in vitro*, while we varied the levels of glucose in the media. Importantly, GLUT3 T cells were more resistant than their MOCK counterparts to cytokine in the absence of glucose over time (**Figure 4F**).

Finally, we sought to evaluate the response of engineered T cells to repeated antigenic stimulation (i.e., stress), using a previously described assay comprising daily stimulation with the SIINFEKL peptide over 5 days which induces exhaustion *in vitro* (28). Expression of PD1 and LAG3 were significantly lower on repetitively stimulated GLUT3- vs. MOCK-T cells, and also TIM3 expression tended to be lower (**Figure 4G**, top). GLUT3-T cells also maintained higher expression levels of TCF1, a transcription factor important in the formation and maintenance of memory (29) (**Figure 4G**, top right). By contrast, there was no difference in expression of the transcription factor TOX, which has been shown to drive exhaustion (30) (**Figure 4G**).

Taken together these data indicated that GLUT3-T cells were less susceptible to cell death and exhaustion than MOCK-T cell counterparts.

### Enforcing expression of GLUT3 by T_CM_ cells enables enhanced tumor control and increased survival of mice

**Figure 5:**
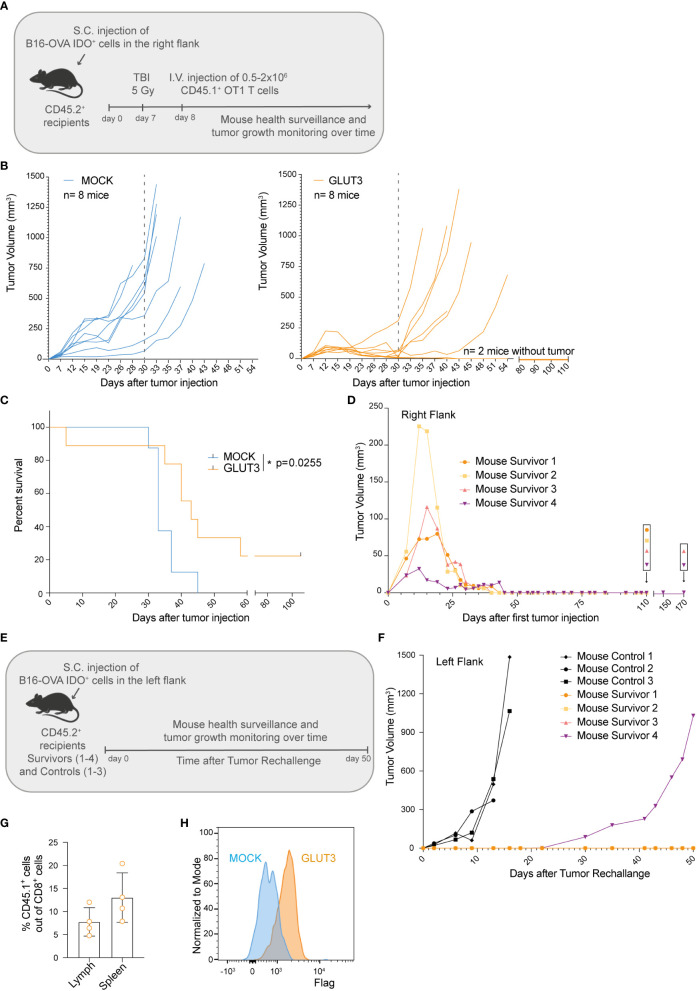
Enforcing expression of GLUT3 by T_CM_ cells enhances tumor control and survival of mice treated by ACT. **(A)** Schematic of adoptive cell transfer (ACT) study. **(B)** Tumor growth over time in individual mice following ACT. Left: ACT of MOCK-T cells. Right: ACT of GLUT3-T cells. Dotted grey line marks 30 days post tumor cells injection. **(C)** Kaplan-Meier analysis of mice survival up to 110 days after tumor cell injection. **(D)** Growth and control of tumors in the right flanks of 4 mice surviving in 2 independent ACT studies. **(E)** Schematic of tumor cell rechallenge experiment in 4 mice survivors. Mouse survivors 1 and 2 (orange dot and yellow square) were newly injected at day 110 post their initial tumor cell injection (previously on their right flanks), and mouse survivors 3 and 4 (pink and purple triangles) at 170 days. **(F)** Tumor growth curves for survivors and control mice. **(G)** T cell persistence in the lymph nodes and spleens of survivors at 50 days post rechallange as assessed by anti-CD45.1 antibody staining and flow cytometric analysis. **(H)** Representative anti-Flag tag antibody staining of T cells extracted from the lymph nodes of survivors. Statistical analysis by Log-rank (Mentel-Cox) **(C)**. *p < 0.05.

To test how superior metabolic and cellular fitness of GLUT3-T_CM_ cells translated to *in vivo* function, B16-OVA IDO^+^ tumor-bearing mice were preconditioned by myeloablative irradiation and then intravenously (I.V.) injected with gene-modified OT1 TCR T cells and monitored over time (**Figure 5A**). The preconditioning was performed to allow higher T cell engraftment (and consequently better tumor control), and also because it more closely mimic patient treatment in the clinic. A significant improvement in tumor control by GLUT3- vs. MOCK- OT1 T cells became apparent around 30 days after tumor cell injection (**Figure 5B**). There was also a significant increase in survival for GLUT3-T cell treated mice, with 25% of the mice alive and apparently cured at the end of the study (**Figures 5C**, **S3A**). We analyzed adoptively transferred CD45.1^+^ tumor infiltrating lymphocytes (TILs) on day 30 post tumor cell injection (**Figure S3B**) but observed no differences in percentage CD45.1 TILs out of CD8^+^ TILs (normalized to tumor weight, **Figure S3C**), nor in their expression levels of the inhibitory markers PD1 and TIM3 (**Figures S3D**, **S3E**) for GLUT3- versus MOCK-OT1 T cells. Upon *in vitro* restimulation, GLUT3-TILs tended to have a higher TMRM/MG ratio (**Figure S3F**), IFNγ (**Figure S3G**), and TNF production (**Figure S3H**) as compared to MOCK-TILs.

Lastly, we sought to determine whether mice that received GLUT3-T cells were protected long-term. To address this question, surviving mice from 2 independent studies (2/8 from each, **Figure 5D**) were re-challenged on the opposite flank (**Figure 5E**) at 110 and 170 days post first tumor cell injection (**Figures 5B**, **S3A**). As a control, previously untreated mice were also injected with tumor cells. Only in 1 of 4 re-challenged mice tumor growth was observed (starting on day 30, **Figure 5F**). At day 50 post-rechallenge the 3 mice were euthanized and autopsy revealed no tumor presence on either flank (representative mouse, **Figure S3I**). Persistence of the transferred GLUT3-T cells was confirmed in both the spleen and draining lymph nodes (axillar and inguinal) of the mice surviving this re-challenge (**Figures 5G, H**). Together these data demonstrated superior tumor control by GLUT3- over MOCK-T cells, enabling cure and protection from rechallenge in a significant proportion of mice.

## Discussion

Glucose is a critical nutrient for the antitumoral activity of T cells as it supports both glycolysis and mitochondrial OXPHOS upon its conversion to pyruvate (31). Several studies have demonstrated that the heavy reliance of tumor cells on glycolysis to meet their metabolic needs can detrimentally impair TILs function by generating a glucose-deprived microenvironment (12, 13, 32). While others have shown that GLUT1 overexpression in T cells promotes glucose uptake, cell size, cytokine production and survival (25, 33), we reasoned that the higher affinity of GLUT3 for glucose, along with its at least five-fold greater transport capacity (17), would confer even greater benefit to engineered T cells. In our study, we demonstrated that enforcing expression of GLUT3 by CD8^+^ T cells increases glucose uptake and drives the accumulation of energy reserves in the form of glycogen and lipids, thereby resulting in a favorable metabolic profile, better resistance to cell death and stress/exhaustion, and superior tumor control upon ACT.

A variety of culture methods, including T cell expansion in the presence of the common gamma chain cytokines IL-7 and IL-15 (26), have been developed to favor a less differentiated phenotype and enhance persistence, a critical parameter for tumor control upon ACT (16). In addition, culture in the presence of PI3K (34) or Akt (35) inhibitors, have been demonstrated to improve T cell fitness allowing better tumor control. Approaches such as these, however, are transient in nature as compared to a gene-engineering strategy like the forced over-expression of proliferator-activated receptor gamma coactivator 1-alpha (PGC-1α; the main regulator of mitochondrial biogenesis) (32, 36). We believe that an important advantage of enforcing GLUT3 expression is that it equips the T cells with an energy reserve allowing superior tumor control. Moreover, we hypothesize that the GLUT3-T cells may be better able to compete for limited glucose supplies in the TME.

Importantly, while enforcing GLUT3 expression in CD8^+^ T cells promoted higher glucose uptake, this metabolic alteration did not induce uncontrolled activation, nor, at the other extreme, exhaustion. Indeed, a link between excessive uptake of glucose by CD4^+^ T cells has been shown to induce hyper-immune activation in the context of HIV-1 infection (37). Whereas, in activated phosphoinositide 3-kinase δ syndrome (APDS) patients, constitutive activation of the PI3K-Akt-mTOR signaling axis causes chronic activation of glycolysis and T cell exhaustion (38, 39). In our study, we observed no evidence of dysregulated activity of GLUT3-T cells, neither *in vitro* nor *in vivo*, and we demonstrated greater resistance of GLUT3-T cells to cell death. Notably, in line with our observations, it was recently demonstrated that GLUT3 overexpression in Th17 cells increases effector function both *in vitro* (cytokine production) and *in vivo* in the context of experimental autoimmune encephalomyelitis (40).

A glucose dependent anti-apoptotic signaling pathway was previously described (25) wherein increased glucose metabolism stabilizes intracellular levels of the Bcl2 family member Mcl-1 which inhibits the pro-apoptotic activity of Bax. This could explain, at least in part, the fact that cancer cells can simultaneously increase their glucose metabolism upon GLUT1 overexpression and their resistance to cell death without growth factors or external stimuli. In accordance, we also observed higher Mcl-1 levels in GLUT3- versus MOCK-T cells. Moreover, by Annexin-V and 7-AAD staining we observed a significantly lower proportion of necrotic GLUT3- than MOCK-T cells in media lacking cytokine support.

In a recent study, it was reported that TILs from melanoma and colon cancer accumulate depolarized mitochondria (as a result of decreased mitophagy) and display characteristics of terminally exhausted T cells (41). Similarly, in a chronic infection model, early exhausted T cells demonstrated impaired glycolytic and mitochondrial metabolism and comprised depolarized mitochondria (42). Moreover, in another study, CD8^+^ TILs from renal cell carcinoma showed decreased uptake of glucose and altered mitochondria, and the authors demonstrated that *in vitro* CD28 costimulation could rescue effector function by increasing glycolysis and consequently mitochondrial potential and mass (9). Here, we have shown that GLUT3-T cells display increased and sustained mitochondrial potential, presumably due to higher levels of glucose uptake, intracellular glucose reserves and energy storage in the form of glycogen and fatty acids.

In an *in vitro* stress assay (i.e., repeated antigenic stimulation) (28), we observed lower upregulation of the inhibitory receptors PD1, LAG3 and TIM3, while maintaining higher levels of the transcription factor TCF1, by GLUT3- versus MOCK-T cells, thus indicating greater resistance to exhaustion. In addition, we observed higher inhibition of GSK-3β in GLUT3-T cells. While this presumably supports glycogen storage by GLUT3-T cells, it may also promote NFAT2 accumulation in the nucleus, T cell proliferation, cytokine production, and memory formation (22, 23). Recently, glycogen metabolism was shown to be critical to the process of memory formation and to sustain anti-tumor immunity (24). Mechanistically, CD8^+^ memory T cells upregulate the expression of phosphoenolpyruvate carboxykinase 1 (Pck1), which enhances carbon allocation into the gluconeogenesis/glycogenolysis circuit generating large amounts of glucose-6-phosphate (G6P). G6P is then oxidized through the pentose phosphate pathway (PPP) to reduced nicotinamide adenine dinucleotide phosphate (NADPH), which reduces glutathione (GSH) and ultimately quenches ROS and supports CD8^+^ T cells memory maintenance and survival. Similarly, we observed lower intracellular ROS levels in GLUT3-T cells. Further characterization of our gene engineered cells to underpin the intricate interplay of GLUT3 overexpression and glycogen metabolism [such as measuring the ratio of reduced glutathione to oxidized glutathione (GSH/GSSG), or of NADP/NADPH] is warranted to elucidate the molecular mechanisms ultimately leading to superior tumor control. Indeed, we observed significantly improved tumor control and survival of mice upon ACT of GLUT3- versus MOCK-T cells. Moreover, a proportion of the mice were cured and protected from tumor rechallenge, indicative of GLUT3-T cell persistence and strong memory recall.

Hence, in summary, we have demonstrated that enforced expression of the glucose transporter GLUT3 supports the metabolic fitness of T cells, renders them more resistant to exhaustion and cell death, and augments their function both *in vitro* and *in vivo*. We conclude that GLUT3-engineering of T cells holds important promise for clinical translation.

## Material and methods

### Generation of retroviral constructs and retrovirus

A gene string encoding GLUT3 and a Flag tag (DYKDDDDK) was purchased from Addgene and cloned in frame in the pMSGV retroviral vector. The pMSGV-Thy1.1 plasmid was used as a control and termed MOCK. Vector amplification was performed in Stellar competent cells (E. coli HST08, 636763, Takara) and purified with plasmid mini/maxi-prep kit (400250/220020, Genomed). Constructs were validated by sequencing (Microsynth AG, Switzerland). Retroviral supernatant was generated as previously described (26).

### Generation and maintenance of tumor cell lines

The B16 melanoma cell line was purchased from the ATCC (ATCC CRL-6475) and gene-modified to express the OVA peptide (SIINFEKL) presented by H-2Kb. For the generation of B16-OVA IDO^+^ cells we generated an amphotropic retroviral pMSGV vector encoding the codon optimized IDO-1 gene linked by T2A to a puromycin resistance gene. Puromycin (540411, Calbiochem) was used at 1 μg/ml to select transduced cells. Wild-type B16, B16-OVA and B16-OVA IDO^+^ cells were kept in culture Dulbecco’s modified eagle medium (DMEM) (3133, Invitrogen) supplemented with 10% heat inactivated (HI; for 30 minutes at 56°C) 10% fetal bovine serum (FBS-26140-079, Gibco) and 1% Penicillin/Streptomycin (PS) and kept at a maximum of 80% confluency.

### Mice

C57BL/6 CD45.2^+^ females were purchased from Harlan Laboratories. OT1 TCR C57BL/6 CD45.1^+^ mice were bred and maintained in-house. All animal experiments were performed in the animal facility in Epalinges at the University of Lausanne (UNIL) under Specific Pathogen Free (SPF) conditions, as approved by the veterinary authorities of the canton of Vaud, and performed in accordance with Swiss Federal Law.

### Splenic T cell isolation, activation, retroviral transduction and expansion

Upon euthanasia of mice with high doses of CO_2_, spleens were harvested and smashed through a 70 μm strainer (352340, Corning) using a 2 ml syringe plunger (01227, Becton Dickinson) and Roswell Park memorial institute medium (RPMI) Glutamax medium (61870010, Invitrogen). Upon red blood cell lysis (43030, BioLegend) of the dissociated splenocytes, T cells were negatively selected using the opportune kit (19851, EasySep Mouse T cells Isolation Kit, Stem Cell) according to the manufacturer’s protocol. Isolated T cells were then resuspended in RPMI Glutamax supplemented with 10% HI FBS, 1% PS, 1% sodium pyruvate (ThermoFisher Scientific, 11360070), 1% non-essential amino acids (MEM non-essential amino acids solution, ThermoFisher Scientific, 11140035), 0.1% 2-mercaptoethanol (ThermoFisher Scientific, 131350010), stimulated with anti-CD3/CD28 dynabeads (2:1=beads: T cells ratio) (11452D, Invitrogen), 50 or 200 IU/ml of human recombinant IL-2 (Glaxo) or murine IL-2 (AF-212-12, Peprotech) and seeded at 1x10^6^ cells/ml in a cell culture treated 24 well plate (CLS3527, Corning). The next day the cells were transduced in a non-treated 24 or 48-well plate (CLS3738, Corning), previously incubated overnight at 4°C with 20 μg/ml of retronectin (T100A Recombinant Human Fibronectin Fragment, Takara), blocked with a solution of phosphate-buffered saline (PBS, 161616, Bischel) with 2% of bovine serum albumin (BSA, A2058, Sigma-Aldrich) for 30 minutes and centrifuged for 90 minutes at 2000 g at 32°C with the retroviral supernatant. Starting from the third day of culture, the cells were maintained at a concentration of 5x10^5^/ml with the addition of human IL-15 (130-093-955, Miltenyi Biotech), human IL-7 (130-093-937, Miltenyi Biotech) at 10 ng/ml or IL-2 200 IU/ml. The T cells were manually counted over time with in a Neubauer chamber (140527, Milian) with trypan blue (15250061, Invitrogen) to distinguish live and dead cells. Alternatively, cells were counted with NucleoCounter^®^200 (Chemometec) counting machine to automatically assess cell number, viability and size (in μM). Population doubling level was calculated as Log (number of cells counted/number of cells previously seeded).

### Extracellular staining of T cells for flow cytometry

T cells were collected in a 96 well V-bottom shaped plate (3849, Corning), washed with Facs Buffer (PBS with the addition of 2% HI FBS and 2 mM of EDTA – 15575020 ThermoFisher Scientific) and incubated with the antibody mixture on ice at 4°C for 30 minutes. The antibodies used were anti-murine CD3 (clone 10A7), anti-murine CD62L phycoerythrin (PE) (clone Mel14, eBioscience), anti- murine CD44 allophycocyanin (APC; clone A20), anti- murine PD1 Brilliant Violent 711 (135231, Biolegend), anti-murine TIM3 Pacific Blue (119723, Biolegend), anti-murine LAG3 PercpCy5.5 (125226, Biolegend), anti-Thy1.1 APC (202526, Biolegend), anti-CD45.1 APC (clone A20). For distinguishing live versus dead cells, the Live/Dead Fixable Aqua dead staining kit (L34957, ThermoFisher Scientific) and Live/Dead fixable near red kit (L10119, ThermoFisher Scientific) were used. The stained samples were kept on ice and acquired with the LSRII or Canto machines at the UNIL Flow Cytometry Facility in Epalinges.

### Intracellular staining of T cells for flow cytometry

The T cells, similarly collected and washed as for extracellular staining, were fixed and permeabilized for 30 minutes at room temperature with the Fix/Perm buffer set kit according to the manufacturer’s protocol (88-8824-00, eBioscience) and stained with the antibody mixture resuspended in permeabilization buffer on ice at 4°C for 30 minutes. The antibodies used were anti-Flag APC (MCA4764A647, Biorad), anti-murine IFNγ PercpCy5.5, anti-murine TNF FITC, Bodipy 493/503 (D3992, Thermofisher), anti-Mcl-1 (12-9047-41, Invitrogen), anti-murine TCF1 (2203S, Bioconcept) and secondary anti-rabbit (4412S, Bioconcept), anti-murine TOX (130-120-716, Miltenyi Biotec GmbH), and secondary anti-rabbit Alexa Fluor 488 (4412, Cell Signaling). For pGSK3b flow staining, the Fix Buffer I (557870, BD), Phospho Flow Perm Buffer III (558050, BD) and anti-phospho-GSK-3β (Ser9) antibody (5558, Cell Signaling) were used according to the manufacturer’s suggestions. For Annexin V/7-AAD staining the Apoptosis Detection kit was used (640930, Biolegend) according to the manufacturer’s protocol. For cell proliferation, the Cell Trace Violent (c34557, Invitrogen) reagent was used according to the manufacturer’s protocol. For mitochondrial staining, Tetramethylrhodamine-Methyl Ester-Perchlorate (TMRM) (T668, Invotrogen) and Mitotracker Green FM (M7514, Invitrogen) were incubated in cell cultures for 30 minutes at 37°C. For oxidative stress detection, Cell ROX Green reagent (C10444, Invitrogen) was used as per manufacturer’s indications. The stained samples were kept on ice and acquired with the LSRII or Canto machines at the UNIL Flow Cytometry Facility.

### Glucose uptake

The fluorescent analogue of glucose (Glc), 2-(N-(7-Nitrobenz-2-oxa-1,3-diazol-4-yl) Amino)-2-Deoxyglucose (2NBDG) was used for the evaluation of glucose uptake. Briefly, 5x10^5^ T cells were incubated for 30 minutes in Glc free media (12633-012, Invitrogen) to normalize the Glc uptake rate across different samples, then incubated with 100 μM of 2NBDG (N13195, Invitrogen) resuspended in 100 μl of Glc free media for 30 minutes at 37°C with the addition or not of 2-DG. Upon extensive washes with PBS, the cells were analyzed with flow cytometry for green fluorescence (FL-1 channel). Data are presented as mean fluorescence intensity (MFI). Alternatively, the Glc uptake luminescence-based assay was used as per the manufacturer’s instructions (J1341, Promega).

### Transmission electron microscopy

The transmission electron microscopy (TEM) pictures were taken at the Electron Microscopy Facility at the UNIL. Briefly, the cells were fixed in glutaraldehyde solution (EMS, Hatfield, PA) 2.5% and in osmium tetroxide 1% (EMS) with 1.5% of potassium ferrocyanide (Sigma, St. Louis, MO) in phosphate buffer (PB 0.1 M [pH 7.4]) for 1 hour at RT. The cells were then washed with H_2_O and embedded in agarose (Sigma, St Louis, MO, US) 2% in H_2_O, dehydrated in acetone solution (Sigma, St Louis, MO, US) at graded concentrations (30%-40 minutes; 70% - 40 minutes; 100% - 2 x 1 hour), infiltrated in Epon resin (EMS, Hatfield, PA, US) at graded concentrations (Epon 33% in acetone-2 hours; Epon 66% in acetone-4 hours; Epon 100%-2x8 hours) and finally polymerized for 48 hours at 60°C in an oven. Ultrathin sections (50 nm thick) were cut using a Leica Ultracut (Leica Mikrosysteme GmbH, Vienna, Austria), picked up on a copper slot grid 2x1 mm (EMS, Hatfield, PA, US) coated with a polystyrene film (Sigma, St Louis, MO, US). The sections were than stained with uranyl acetate (Sigma, St Louis, MO, US) 4% in H_2_O for 10 minutes, rinsed several times with H_2_O followed by Reynolds lead citrate in H_2_O (Sigma, St Louis, MO, US) for 10 minutes and rinsed several times with H_2_O. Micrographs were taken with a TEM FEI CM100 (FEI, Eindhoven, The Netherlands) at an acceleration voltage of 80kV with a TVIPS TemCamF416 digital camera (TVIPS GmbH, Gauting, Germany).

### Glycogen and glucose quantification

Intracellular glycogen levels were measured using the glycogen colorimetric assay kit (K648, Biovision) following the manufacturer’s instructions. Briefly, cells were homogenized in H_2_O on ice, boiled for 10 minutes, spun at 18000 g for 10 minutes and supernatants were analyzed for glycogen content. Results were normalized by cell number. For intracellular glucose content in T cells, the background values obtained with the glycogen colorimetric assay kit (K648, Biovision) are shown.

### Periodic acid schiff staining

A suspension of 1-5x10^5^ T cells was seeded on a poly-L Lysine slides, fixed with 4% paraformaldehyde, incubated in solution of periodic acid (100524, Merck) 1% for 5 minutes at room temperature, rinsed in distilled H_2_O and dipped in Schiff reagent (109033, Merck) for 20 minutes. The slides were then stained with Harris’s Hematoxylin (HHS16, Sigma Aldrich). The control slides were treated with a solution of amylase 0.1% (10080, Fluka) for 30 minutes at 37°C prior to the staining. The stainings were performed at the Pathology Institute of the UNIL. The slides were scanned with a Nanozoomer Slide Scanner at 40X magnification with NDP view 2 software. The pictures were analyzed with a customized macro (Area/min and max gray value/Mean Gray value/Limit to threshold/display label/Add to overlay) applied on FIJI software.

### T cell *in vitro* assays

After 9-12 days of culture, T cells were cultured in Glc-free media (12633-012, Invitrogen or DMEM (D5030, Sigma) supplemented with 10% HI dialyzed FBS (A3382001, Invitrogen), 1% P/S, 1% sodium pyruvate (ThermoFisher Scientific, 11360070), 0.1% 2-mercaptoethanol (ThermoFisher Scientific, 131350010) with the addition or not of D-Glc (G-8270, Sigma) at 0.444 mM (referred as low Glc) concentration or 11.1mM (referred as complete media).

For cytokine production analysis, the T cells were stimulated overnight with 0.1 μg/ml of SIINFEKL peptide (from the Protein Peptide Chemistry Facility at UNIL) and analyzed by flow cytometry. For proliferation assessment, B16-OVA melanoma tumor cells were lethally irradiated with 33 Gray (Gy) with a 137 Caesium source irradiator (LISA-1) according to UNIL safety protocols and seeded at a 1:1 ratio with CTV prelabeled T cells for 5 days.

For exhaustion assessment, T cells were stimulated with 10 ng/ml of SIINFEKL peptide every day for 5 days following a previously published protocol (28). For mitochondrial analysis, cells were incubated for 30 minutes at 37°C with 2 μM of TMRM and 100 nM of Mitotracker Green (MG). Where indicated the cells were pretreated overnight with 100 μM 2-DG (D8375, Sigma Aldrich).

### Adoptive T cell transfer experiments

Briefly, 1x10^5^ B16-OVA IDO^+^ cells were subcutaneously injected in the right flank mice aged 8 weeks. Once the tumor was palpable after 7 days the mice received 5 Gy irradiation and the following day an intravenous injection of 0.5-2x10^6^ T cells. For rechallenge experiments, 1x10^5^ B16-OVA IDO^+^ cells were subcutaneously injected in the left flank of the mice. During experimentation, all mice were monitored at least every other day for signs of distress. Mice were euthanized at end-point by CO_2_ and, where indicated, tumors, spleens and peripheral blood were collected. For *ex vivo* analysis, at terminal point solid tumor mass was excised from the mice, weighed, cut into small pieces with a scalpel, passed through 70 µm pore cell strainers (Grenier Bio-One) and centrifuged for 5 minutes at 1500 rpm to pellet the cells. The cells were then resuspended in 3 ml of PBS and a Ficoll gradient (CL5035, Cederlane) was performed to eliminate dead cells and tumoral debris by centrifugation at 2000 rpm for 20 minutes with acceleration 2 and brake 7. Upon extensive washes, the cells were used for flow cytometric analysis and *in vitro* restimulation with SIINFEKL peptide.

### Statistical analysis

All the statistical analysis were performed with GraphPad Prism 9.0 (GraphPad Software, La Jolla, CA). Starting from p< 0.05 the difference was considered significant.

## Data availability statement

The raw data supporting the conclusions of this article will be made available by the authors, without undue reservation.

## Ethics statement

The animal study was reviewed and approved by Swiss Veterinary Authorities (SCAV).

## Author contributions

MI and GC directed the study. EC, GMPGA, and PG designed and performed experiments. AS-G performed experiments. CH, NV, and RVS provided expert feedback. EC, GMPGA, PG, and MI analyzed data. EC and MI wrote the manuscript. All authors contributed to the article and approved the submitted version.

## Funding

This work was generously supported by Ludwig Institute for Cancer Research and the Swiss National Science Foundation (to MI: 310030_204326). The Prostate Cancer Foundation and Cancera.

## Conflict of interest

The authors declare that the research was conducted in the absence of any commercial or financial relationships that could be construed as a potential conflict of interest.

## Publisher’s note

All claims expressed in this article are solely those of the authors and do not necessarily represent those of their affiliated organizations, or those of the publisher, the editors and the reviewers. Any product that may be evaluated in this article, or claim that may be made by its manufacturer, is not guaranteed or endorsed by the publisher.

## Supplementary material

Supplementary Figure 1 Characterization of MOCK- and GLUT3-T_EM_ cells. **(A)** Analysis of Thy1.1 expression by transduced MOCK-T_EM_ cells. **(B)** Representative 40X pictures of PAS-stained MOCK- and GLUT3-T cells. Red arrows indicate PAS positive cells, blue arrows indicate PAS negative cells. **(C)** PAS staining slides treated with amylase, used as negative control. **(D)** Representative dot plot of TMRM and MG stained cells incubated overnight with or without 2-DG.

Supplementary Figure 2 Characterization of MOCK- and GLUT3-T_CM_ cells. **(A)** Analysis of Thy1.1 expression in transduced MOCK-T_CM_ cells. **(B)** MOCK- and GLUT3-T_CM_ cell size (μm). **(C)** Population doubling of MOCK- and GLUT3-T_CM_ cells over time. **(D)** Left: glucose uptake evaluated with the fluorescent glucose analog 2-NBDG with or without the addition of the glucose competitor 2-DG7. Right: representative histograms of 2-NBDG uptake. **(E)** Glucose uptake by T cells as measured with a luminescence-based assay. Shown is average ± SD of different cultures. Statistical analysis by paired, two-tailed *t* test **(B, E, F)**, unpaired, two-tailed *t* test (**D**: MOCK vs GLUT3 p=0.009, MOCK vs MOCK+2DG p= 0.0493, GLUT3 vs a GLUT3+2DG p=0.0016).

Supplementary Figure 3
*In vivo* evaluation of MOCK- and GLUT3-T cells against B16-OVA IDO^+^ tumors. **(A)** Tumor growth curves in individual mice upon ACT in an second independent study. Left: MOCK-T cell ACT. Right: GLUT3-T cell ACT with 2 surviving mice. **(B)** Schematic of ACT studies for *ex vivo* analyses of tumor infiltrating lymphocytes (TILs). **(C)** Percentage of CD45.1^+^ TILs in CD8^+^ TILs normalized to tumor volume **(D)** Expression levels (MFI) of PD1 and **(E)** TIM3 by TILs. **(F)** Left: analysis of TMRM and MG staining of TILs, expressed as ratio between TMRM MFI geometric mean (GeoMean) versus MG MFI GeoMean. Right: representative dot plot of TMRM/MG staining of TILs (right panel). **(G, H)** Analysis of IFNγ and TNF expression from isolated TILs upon overnight stimulation with SIINFEKL peptide. Data are pooled from 2 independent studies. **(I)** Picture of a survivor upon euthanasia. Arrows indicate the sites of tumor cell injection on the right and left flanks. Statistical analysis by unpaired, two-tailed *t* test **(F)**. **p< 0.01; *p < 0.05.

## Abbreviations

ACT, adoptive T cell therapy; TME, tumor microenvironment; TIL, tumor infiltrating lymphocyte; GLUT3, Glucose Transporter 3.
