# Supplementary figures and images for: Enforcing GLUT3 expression in CD8+ T cells improves fitness and tumor control by promoting glucose uptake and energy storage
Source: Front Immunol. 2022 Sep 20;13:976628. doi: 10.3389/fimmu.2022.976628 (PMC9530831; doi:10.3389/fimmu.2022.976628)

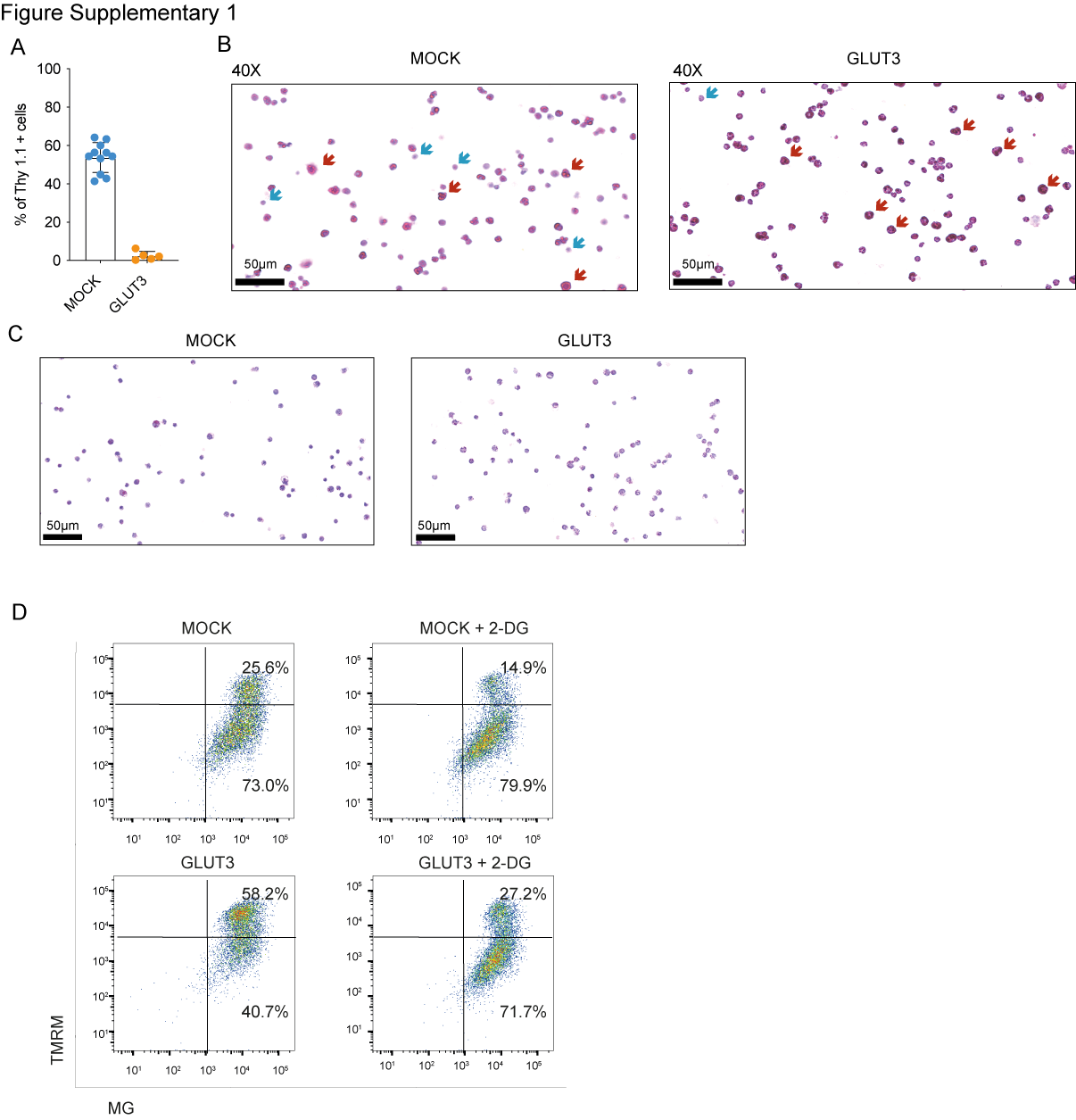

Supplement: Supplementary Figure 1 — Characterization of MOCK- and GLUT3-TEM cells. (A) Analysis of Thy1.1 expression by transduced MOCK-TEM cells. (B) Representative 40X pictures of PAS-stained MOCK- and GLUT3-T cells. Red arrows indicate PAS positive cells, blue arrows indicate PAS negative cells. (C) PAS staining slides treated with amylase, used as negative control. (D) Representative dot plot of TMRM and MG stained cells incubated overnight with or without 2-DG. [file Image_1.tif]

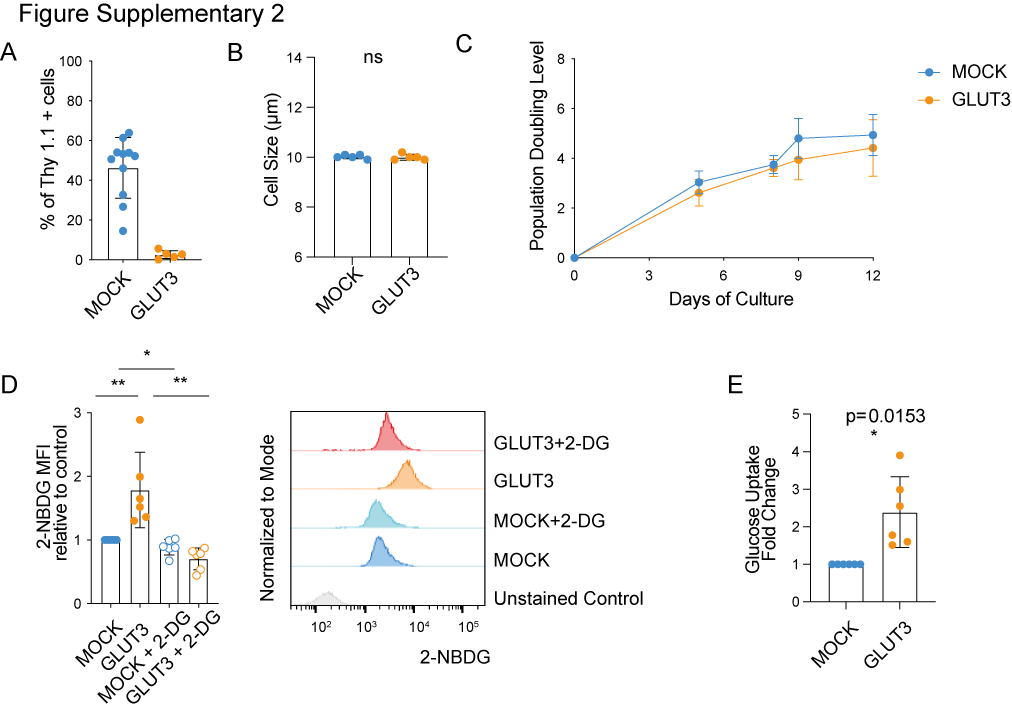

Supplement: Supplementary Figure 2 — Characterization of MOCK- and GLUT3-TCM cells. (A) Analysis of Thy1.1 expression in transduced MOCK-TCM cells. (B) MOCK- and GLUT3-TCM cell size (μm). (C) Population doubling of MOCK- and GLUT3-TCM cells over time. (D) Left: glucose uptake evaluated with the fluorescent glucose analog 2-NBDG with or without the addition of the glucose competitor 2-DG7. Right: representative histograms of 2-NBDG uptake. (E) Glucose uptake by T cells as measured with a luminescence-based assay. Shown is average ± SD of different cultures. Statistical analysis by paired, two-tailed t test (B, E, F), unpaired, two-tailed t test (D: MOCK vs GLUT3 p=0.009, MOCK vs MOCK+2DG p= 0.0493, GLUT3 vs a GLUT3+2DG p=0.0016). [file Image_2.tif]

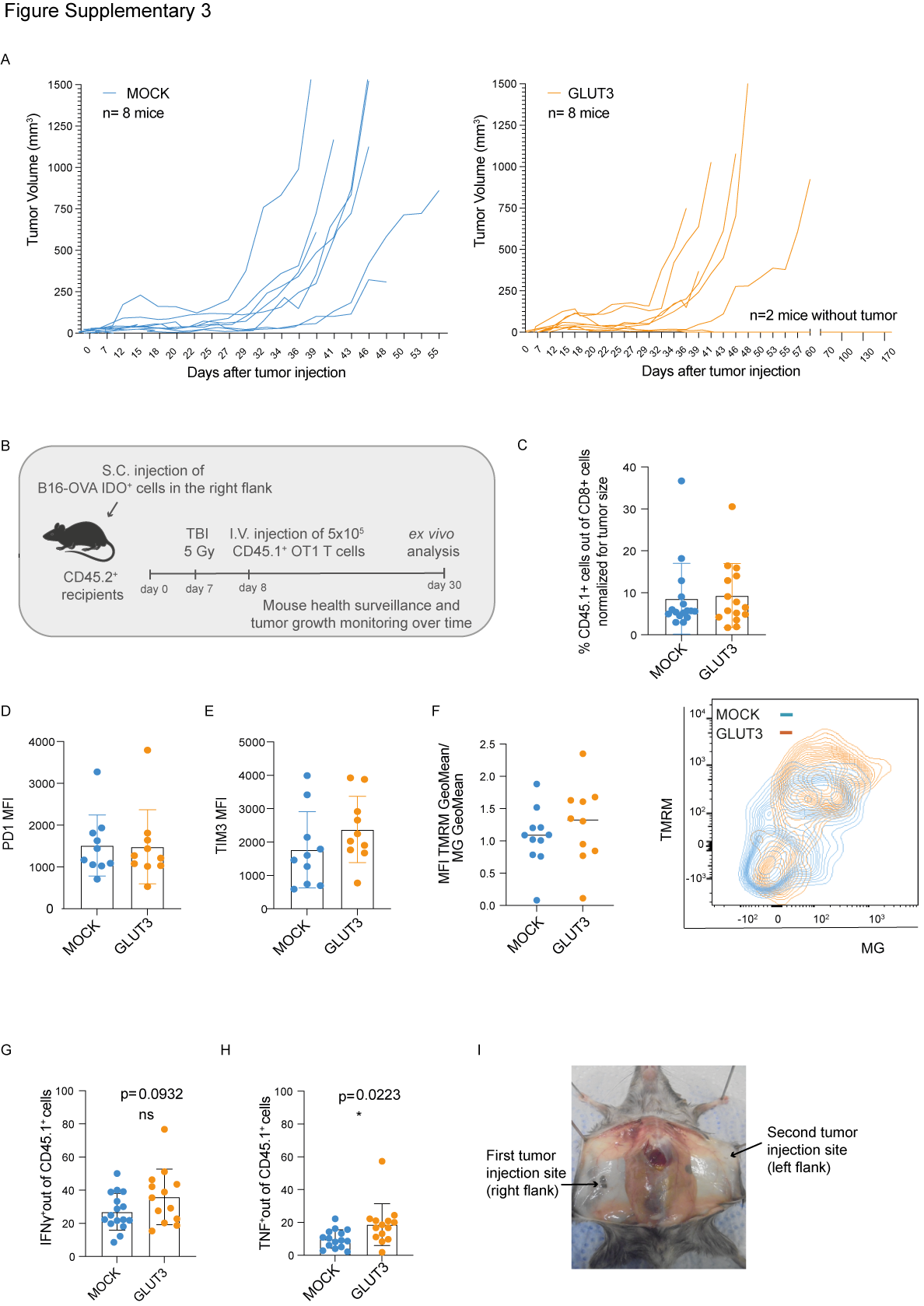

Supplement: Supplementary Figure 3 — In vivo evaluation of MOCK- and GLUT3-T cells against B16-OVA IDO+ tumors. (A) Tumor growth curves in individual mice upon ACT in an second independent study. Left: MOCK-T cell ACT. Right: GLUT3-T cell ACT with 2 surviving mice. (B) Schematic of ACT studies for ex vivo analyses of tumor infiltrating lymphocytes (TILs). (C) Percentage of CD45.1+ TILs in CD8+ TILs normalized to tumor volume (D) Expression levels (MFI) of PD1 and (E) TIM3 by TILs. (F) Left: analysis of TMRM and MG staining of TILs, expressed as ratio between TMRM MFI geometric mean (GeoMean) versus MG MFI GeoMean. Right: representative dot plot of TMRM/MG staining of TILs (right panel). (G, H) Analysis of IFNγ and TNF expression from isolated TILs upon overnight stimulation with SIINFEKL peptide. Data are pooled from 2 independent studies. (I) Picture of a survivor upon euthanasia. Arrows indicate the sites of tumor cell injection on the right and left flanks. Statistical analysis by unpaired, two-tailed t test (F). **p< 0.01; *p < 0.05. [file Image_3.tif]
